# Honeybees affect floral microbiome composition in a central food source for wild pollinators in boreal ecosystems

**DOI:** 10.1007/s00442-022-05285-7

**Published:** 2022-11-24

**Authors:** Elsi Hietaranta, Heli Juottonen, Minna-Maarit Kytöviita

**Affiliations:** Department of Biological and Environmental Science, University of Jyväskylä, P.O. Box 35, 40014 Jyväskylä, Finland

**Keywords:** *Salix phylicifolia*, Inflorescence, Bacteria, Fungi, Richness

## Abstract

Basic knowledge on dispersal of microbes in pollinator networks is essential for plant, insect, and microbial ecology. Thorough understanding of the ecological consequences of honeybee farming on these complex plant–pollinator–microbe interactions is a prerequisite for sustainable honeybee keeping. Most research on plant–pollinator–microbe interactions have focused on temperate agricultural systems. Therefore, information on a wild plant that is a seasonal bottleneck for pollinators in cold climate such as *Salix phylicifolia* is of specific importance. We investigated how floral visitation by insects influences the community structure of bacteria and fungi in *Salix phylicifolia* inflorescences under natural conditions. Insect visitors were experimentally excluded with net bags. We analyzed the microbiome and measured pollen removal in open and bagged inflorescences in sites where honeybees were foraging and in sites without honeybees. Site and plant individual explained most of the variation in floral microbial communities. Insect visitation and honeybees had a smaller but significant effect on the community composition of microbes. Honeybees had a specific effect on the inflorescence microbiome and, e.g., increased the relative abundance of operational taxonomic units (OTUs) from the bacterial order Lactobacillales. Site had a significant effect on the amount of pollen removed from inflorescences but this was not due to honeybees. Insect visitors increased bacterial and especially fungal OTU richness in the inflorescences. Pollinator visits explained 38% variation in fungal richness, but only 10% in bacterial richness. Our work shows that honeybee farming affects the floral microbiome in a wild plant in rural boreal ecosystems.

**Supplementary Information:**

The online version contains supplementary material available at 10.1007/s00442-022-05285-7.

## Introduction

Despite their relatively short lifespan, flowers are associated with a rich community of fungi and bacteria, i.e., they contain a diverse microbiome (Alvarez-Perez et al. 2012; Junker and Keller 2015; Manirajan et al. 2016; Shade et al. 2013). The microbiome of floral surfaces and nectar is a result of interacting biotic and abiotic factors. One of the important biotic factors affecting floral microbiome is insect visitors of flowers. Although microbes are already present on flowers that have not been visited, horizontal transmission of microbes to and among flowers by insect visitors shapes the floral microbiome composition (Morris et al. 2020; Vannette and Fukami 2017; de Vega and Herrera 2013). Pollinators have been shown to be important vectors of both the bacterial (Allard et al. 2018; Ushio et al 2015) and fungal component (Belisle et al. 2012; Brysch-Herzberg 2004; Herrera et al. 2010, 2009; Pozo et al. 2012; de Vega et al. 2009) of floral microbiome.

Flower-visiting insects have been shown to vector microbes in a species-specific manner (Brysch-Herzberg 2004; Herrera et al. 2009; Lachance et al. 2001; Morris et al. 2020; Ushio et al. 2015; de Vega et al. 2009; de Vega and Herrera 2013). Consequently, insect species-specific surface microbes remaining on a flower surface could be used as “fingerprint” to identify candidate pollinator species for the plant (Ushio et al. 2015). Honeybees have been shown to affect the floral microbiomes in crop plants (Aizenberg-Gershtein et al. 2013). Honeybee farming in Europe is intensive, and currently, there are about 16 million beehives in Europe with 72,300 beehives in Finland (Finnish Beekeeping Program 2018), 125,000 beehives in Sweden (Chauzat et al. 2013), and 50,000 beehives in Norway (Chauzat et al. 2013). Bee farming is particularly intensive in rural areas where uncropped farmland provides floral resources for honey production.

Honeybee-vectored microbes may have various ecological consequences. Pathogen spill-over from managed bees to native wild pollinators is of particular concern worldwide (Fürst et al. 2014; Graystock et al. 2015; Koch et al. 2017). Pathogen transmission through flowers involves also both animal (Durrer and Schmid-Hempel 1994) and plant pathogens (Alexandrova et al. 2002). Pollinators may vector microbes that are not beneficial to the plant such as sexually transmitted diseases (McArt et al. 2014; Proesmans et al. 2021). Several floral microbes are known to be pathogenic to plants (Spanos and Woodward 1994) and can reduce plant fitness (Alexander and Antonovics 1988). The specific effect of honeybees on floral microbiomes of wild plants has not been investigated previously despite the large potential of honeybees vectoring microbes outside agricultural systems.

In addition to insect visitors, a few studies have shown that plant species (von Arx et al. 2019; Aizenberg-Gershtein et al. 2013; Fridman et al. 2012; Wei and Ashman 2018) and within species the plant individual affects plant (Wagner et al. 2016; Peiffer et al. 2013) and floral (Boachon et al. 2019) microbiome. The genotype has a great influence on the plant secondary chemistry (Laitinen et al. 2005) which is known to affect plant microbiome (Cotton et al. 2019; Huang et al. 2019). Plant secondary chemistry and genotype have been shown to affect *Arabidopsis thaliana* floral microbiome under growth chamber conditions (Boachon et al. 2019). However, the relative role of plant individual in defining floral microbiome composition under natural conditions has not been evaluated previously. In addition to biotic factors, abiotic conditions, such as season (von Arx et al. 2019), wind (Shade et al. 2013), temperature (Herrera and Pozo 2010; Pusey and Curry 2004; Baruzzi et al. 2012), and UV radiation (Figueroa et al. 2019), may have an impact. Yet, the relative importance of the ecological drivers such as insect visitors and abiotic factors that shape the floral microbiome are largely unquantified.

Many studies of insect visitation on floral microbiome have been conducted in temperate climate (Brysch-Herzberg 2004; Pozo et al. 2012; de Vega and Herrera 2013) or on crop plants (Aizenberg-Gershtein et al. 2013; Fürnkranz et al. 2012; Vannette et al. 2017). Information on factors regulating microbiome of cultivated species may not translate directly to wild plants (Pérez-Jaramillo et al. 2016) due to the selection during domestication (Soldan et al. 2021). Studies have also been conducted on non-crop plants but mainly in Mediterranean or dry climates (Rebolleda Gómez and Ashman 2019; Schaeffer et al. 2015; Vannette and Fukami 2018). Therefore, knowledge on a wild plant that is a seasonal bottleneck for pollinators in cold climate is of specific importance.

*Salix phylicifolia* (tea-leaved willow) is a particularly important food source for wild pollinators (Alford 1975; Elmqvist et al. 1988) in the north, because in early spring, other floral resources are scarce and male *Salix phylicifolia* provides both nectar and pollen. In addition, the simple structure of the willow inflorescences and large blooms when few other species flower make *Salix phylicifolia* a temporal hub for plant–insect networks (Proesmans et al. 2021). Pollen collected from the male willow inflorescences is a rich source of protein (Roulston and Cane 2000) and vital to the development of insect larvae (Chen 1966). Nectar, on the other hand, provides sugars, amino acids, and fatty acids necessary to sustain active adult insects (Baker and Baker 1986, 1973).

Here, we investigated in a manipulative experiment how floral visitation by insects, plant individual, and site influence the community structure of bacteria and fungi in *Salix phylicifolia* inflorescences in boreal ecosystems. We asked the following questions (i) Do cultivated honeybees affect *Salix phylicifolia* inflorescence microbiome? (ii) What is the relative importance of insect visitation compared to environmental factors and plant individual on microbial community composition in *Salix phylicifolia* inflorescences? (iii) Is bacterial and fungal richness equally affected by insect visitors? (iv) Is pollen removal related to inflorescence microbiome changes? To answer these questions, we analyzed the microbiome and measured pollen removal in open and bagged inflorescences in wild individual *Salix phylicifolia* plants in sites where honeybees were foraging and in sites without honeybees. We used pollen removal from inflorescences as an indirect measure of insect visitation intensity.

We hypothesized that honeybees will have a specific effect on inflorescence microbiome and that plant individual will affect inflorescence microbiome composition. We also hypothesized that floral visitation by pollinators will change microbial community composition and increase microbial richness in inflorescences. Finally, it is not known whether the effect of pollinators on the relative dispersal of bacteria and fungi to flowers differs, because only a few studies survey both groups of microbes (but see Morris et al. 2020; Ottesen et al. 2013; Vannette and Fukami 2017) and few inspect the entire flower (but see Alekett et al. 2014, Junker and Keller 2015; Pozo et al. 2012; Russell et al. 2019). Pollen is removed from flowers when insects visit flowers and forage for nectar and pollen. Theoretically, the more insects forage in a given flower, the more pollen is removed and the more contact between the flower and the insects there is. Therefore, we hypothesized that pollen removal increases microbial diversity in inflorescences.

## Materials and methods

### Study sites and experimental design

The study was conducted in six rural sites in central Finland (Table S1). Three of the study sites located near an apiary (mean distance 100 m, range 10–200 m) and the other three were in areas where honeybees were known from our previous field work to be absent. We selected four male willow (*Salix phylicifolia*) plants in each study site. The selected plants were distinct individuals and thus represented different genotypes within circa one hectare study area. For each willow plant, we selected four similar branches and allocated them into two categories: natural visitation of inflorescences by insects (‘open’) and exclusion of insect visits by a net bag (‘bagged’). For each bagged branch, the distal part with multiple catkins (unopened inflorescences of willow) was enclosed with a net bag (1 mm × 1 mm mesh) at the end of April 2019 (Fig. S1). The weather conditions were favorable for insect visitations, it was sunny, and there was no precipitation during the flowering period. At the peak of the flowering, 5–13 days after bagging, insect visitations on willow inflorescences were observed for about 30 min to verify the presence of wild pollinators and the presence/absence of honeybees in the study sites. Bumblebees were the most common wild insect visitors in the inflorescences. After visitation assessment, 2–3 inflorescences per branch were collected as a pooled sample. Thus, 16 samples (4 pooled inflorescence samples from 4 plants) in each of the 6 study sites were collected (in total 96 samples). Samples were stored at + 4 °C and processed within 24 h.

### Pollen counts and removal by pollinators

In laboratory, each pooled inflorescence sample was briefly shaken in 15 ml of sterilized 0.1% Tween 20® in 0.15 M NaCl. Then, surface microbes were detached by ultrasonic dispersion for 20 s at maximum power (Ultrasonic Cleaner, VWR® International). After the detachment, 200 µl of the solution was taken for pollen particle count and the rest was filtered on a polycarbonate filter membrane (0.2 µm pores, Ø25 mm, Millipore, Billerica, MA, USA). Finally, the sample was rinsed with an additional 5 ml of the Tween-NaCl solution, briefly shaken and filtered on the same filter membrane. The membrane was stored at – 80 °C until DNA extraction. As a control for microbial contamination, Tween-NaCl solution without a sample was filtered as the first and the final filtration. One sample was lost during processing resulting in 95 samples at the end.

To count the amount of pollen in the inflorescences, we analyzed pollen concentration in the Tween-NaCl solution used for microbiome analysis with Casy TT Cell counter (Omni Life Sciences GmbH) as an average of three replicate measurements using the 60 µm capillary, 10 ml of Casy solution, and 10 µl of sample. We calculated the pollen remaining in the inflorescences after insect visitation as the difference between the open and bagged inflorescences divided by the value in bagged inflorescences within the same branch.

### DNA extraction, PCR, and sequencing

DNA was extracted from filter membrane using the NucleoSpin® Soil kit (Macherey‐Nagel, Düren, Germany). Sample lysis was carried out by bead beating at 5.0 m/s for two 45-s cycles (OMNI Bead Ruptor Elite, OMNI International, USA) with two 3.2-mm stainless steel beads and 0.1-mm glass beads in lysis buffer (SL1). DNAs were stored at – 80 °C until further processing. A blank control extraction without a filter membrane was carried out before and after the sample extractions.

Amplification of the bacterial 16S rRNA gene was conducted as nested PCR to limit co-amplification of plant chloroplasts and mitochondria. The first PCR step for the V6–V8 region was carried out with primers 799F (5′-AACMGGATTAGATACCCKG-3′) and 1492R (5′-GGYTACCTTGTTACGACTT-3′) (Chelius and Triplett 2001). A 25-μl PCR reaction contained 0.2 mM of dNTPs, 0.24 μM of each primer, and 0.75 U of DNA polymerase (GoTaq, Promega) in 1 × reaction buffer and 1 μl of extracted DNA as template (12.5–55 ng). The reaction conditions were as follows: an initial denaturation at 95 °C for 3 min, 22 cycles (95 °C, 30 s; 53 °C, 40 s; 72 °C, 60 s) and a final elongation of 72 °C for 5 min. The PCR product served as a template in the second step of the nested PCR with the primers M13-1062F (M13 linker for attaching barcodes and sequencing adapters 5′-TGTAAAACGACGGCCAGT-3′ followed by 1062F 5′-GTCAGCTCGTGYYGTGAG-3′) (Allen et al. 2005; Ghyselinck et al. 2013) and 1390R (5′-ACGGGCGGTGTGTRCAA-3′) (Zheng et al. 1996) using the same reaction contents and conditions as in the first step except 20 cycles.

Amplification of the fungal ITS2 region (intergenic transcribed spacer) was conducted with primers M13-fITS7 (M13 linker 5′-TGTAAAACGACGGCCAGT-3′ followed by fITS7 5′-GTGARTCATCGAATCTTTG-3′) and ITS4 (5′-TCCTCCGCTTATTGATATGC-3′) (Ihrmark et al. 2012). A 25-μl reaction contained 0.4 μM of each primer; otherwise, the composition of the PCR reaction was the same as in amplification of bacteria. The PCR was performed as follows: an initial denaturation at 94 °C for 3 min, 24 cycles (94 °C, 30 s; 55 °C, 30 s; 72 °C, 30 s) and a final elongation of 72 °C for 5 min.

Barcodes and Ion Torrent sequencing adapters were added to the bacterial and fungal amplifications in a separate PCR step with 8 cycles, where forward primer included IonA sequencing adapter, barcode, and M13 linker. Reverse primer contained the 1390R (bacteria) or ITS4 (fungi) primer sequence and adapter P1. PCR products were purified using the AMPure XP beads (Beckman Coulter, Life Sciences) and quantified using Quant-iT™ PicoGreen® dsDNA Assay (Molecular Probes, Eugene, OR). Equal amounts of PCR products were pooled for sequencing. The 16S rRNA gene products were pooled based on the estimated concentration of the bacterial product (ca. 350 bp) from analysis of gel pictures with software ImageJ, and after pooling separated from the plant mitochondrial product (ca. 700 bp) by gel extraction (Monarch® DNA Gel Extraction Kit (BioLabs Inc., New England)). Libraries were sequenced on Ion Torrent PGM using Ion PGM Hi-Q View OT2 Kit, PGM Hi-Q View Sequencing Kit and Ion 316™ Chip v2 (Life Technologies, USA).

### Sequence data processing

The 16S rRNA gene and fungal ITS sequences were processed in mothur v.1.43 (Schloss et al. 2009) following the relevant parts of the MiSeq SOP outlined below (https://mothur.org/wiki/MiSeq_SOP, accessed in April 2020; Kozich et al. 2013). Sequences were quality filtered using average quality of 20 and a window size of 10 bases, a minimum sequence length of 200 bp, a maximum length of 400 bp for bacteria and 410 bp for fungi, maximum homopolymer length = 8, maximum number of ambiguous bases = 0, maximum number of differences to primer sequence = 1, and maximum number of differences to barcode sequence = 0. Fungal ITS2 region was extracted from ITS amplicons with the ITSx software (v. 1.1.2, Bengtsson-Palme et al. 2013). Bacterial sequences were aligned against the Silva database v.1.38 (Quast et al. 2013). Chimeras were detected with command chimera.vsearch with setting dereplicate = T. After quality filtering, alignment (for bacteria), and removal of chimeras and nontarget sequences, there were 764 923 bacterial sequence reads and 404 685 fungal reads. Reads were preclustered with setting diffs = 2 for bacteria and diffs = 1 for fungi. The sequences were clustered into operational taxonomic units (OTUs) using the opticlust method for bacteria and agc for fungi and 97% cutoff for both. 16S rRNA gene OTUs were classified in mothur against the SILVA v.1.38 database and fungal ITS OTUs against the Unite database (v. 8.2, Abarenkov et al. 2020). The sequence data were submitted to NCBI under BioProject accession PRJNA776874.

### Statistical analysis

For microbial community analyses, R (v. 4.0.3, R Core Team 2014) and RStudio with packages ‘vegan’ (v. 2.5–6, Oksanen et al. 2019) and ‘phyloseq’ (McMurdie and Holmes 2013) were used. Singleton OTUs were removed from the dataset. The median number of reads (bacteria 5198, fungi 3260 reads) were selected from the samples with the function rrarefy in vegan and samples with less than the median number of reads were included as such. We compared bacterial and fungal communities between open and bagged inflorescences, study sites, plant individuals, and presence/absence of honeybees using permutational multivariate analysis of variance (PERMANOVA, McArdle and Andersson 2001) and the ‘adonis2’ function in vegan based on Bray–Curtis dissimilarities, 999 permutations, and separate models for each factor. In the tests for the effect of bagging and plant individual, site was used as a blocking factor. The effect of the plant individual was also analyzed separately for each site. The effect of honeybees was tested with a model including only the open inflorescences in all sites. Finally, we examined which OTUs were affected by bagging and the presence of honeybees using differential abundance analysis (DESeq2) (Love et al. 2014). When testing the effect of honeybees, only the open inflorescences were included. In this analysis, OTU data were not rarefied and only OTUs with 48 or more reads were included to represent OTUs occurring consistently in at least one sample type. The OTUs with log2 fold change > 1 or < − 1 and adjusted *p* value < 0.05 were considered affected by the treatments. The R code and the data files for the analyses are provided at https://github.com/helijuottonen/elsiwillow.

Microbial richness (the number of OTUs) and pollen data were analyzed with the statistical software PASW 18.0 (IBM SPSS Statistics). The relative importance of different explanatory factors for microbial richness was analyzed using nested analysis of variance (nested ANOVA). Honeybees could not be included in the analysis as beehives were either present or not present in each site, but bagging, individual, and site were included as fixed factors. We also compared whether pollen counts between samples from open and bagged inflorescences differed using ANOVA. The samples had one outlying value, and therefore, the data were log transformed. The effect of honeybees on pollen removal was analyzed with Mann–Whitney U test, since the data were not normally distributed.

## Results

### Microbial community composition

Presence of honeybees affected significantly the community composition of both bacteria and fungi on the inflorescences (Table 1). Compared to honeybees, bagging, i.e., excluding insect visitors, explained a slightly smaller amount of the variation in bacterial and fungal communities. The most important factors explaining *Salix phylicifolia* inflorescence microbiome composition were the study site and plant individual (Table 1, Table S2).

**Table 1:** PERMANOVA statistics on the factors that explain bacterial and fungal community composition in *Salix phylicifolia* inflorescences

Factors	Bacteria					Fungi				
	*df*	*SS*	*R*^*2*^	*F*	*p*	*df*	*SS*	*R*^*2*^	*F*	*p*
Bagging	1	0.44	0.02	1.85	0.048*	1	0.43	0.02	2.05	0.002**
Honeybees	1	0.56	0.05	2.23	0.044*	1	0.57	0.06	2.76	0.002**
Site	5	3.38	0.15	3.12	0.001***	5	4.76	0.24	5.60	0.001***
Individual	23	10.6	0.47	2.70	0.001***	23	10.4	0.52	3.36	0.001***

‘Bagging’ refers to the effect of bagging inflorescences (open/bagged), factor ‘Honeybees’ refers to the presence/absence of honeybees in the sites, factor ‘Site’ refers to the six study sites (see Table S1), and factor ‘Individual’ refers to the four plant individuals in each study site. Significance codes are *p* ≤ 0.001 = ***, *p* ≤ 0.01 = **, *p* ≤ 0.05 = *

**Fig. 1:**
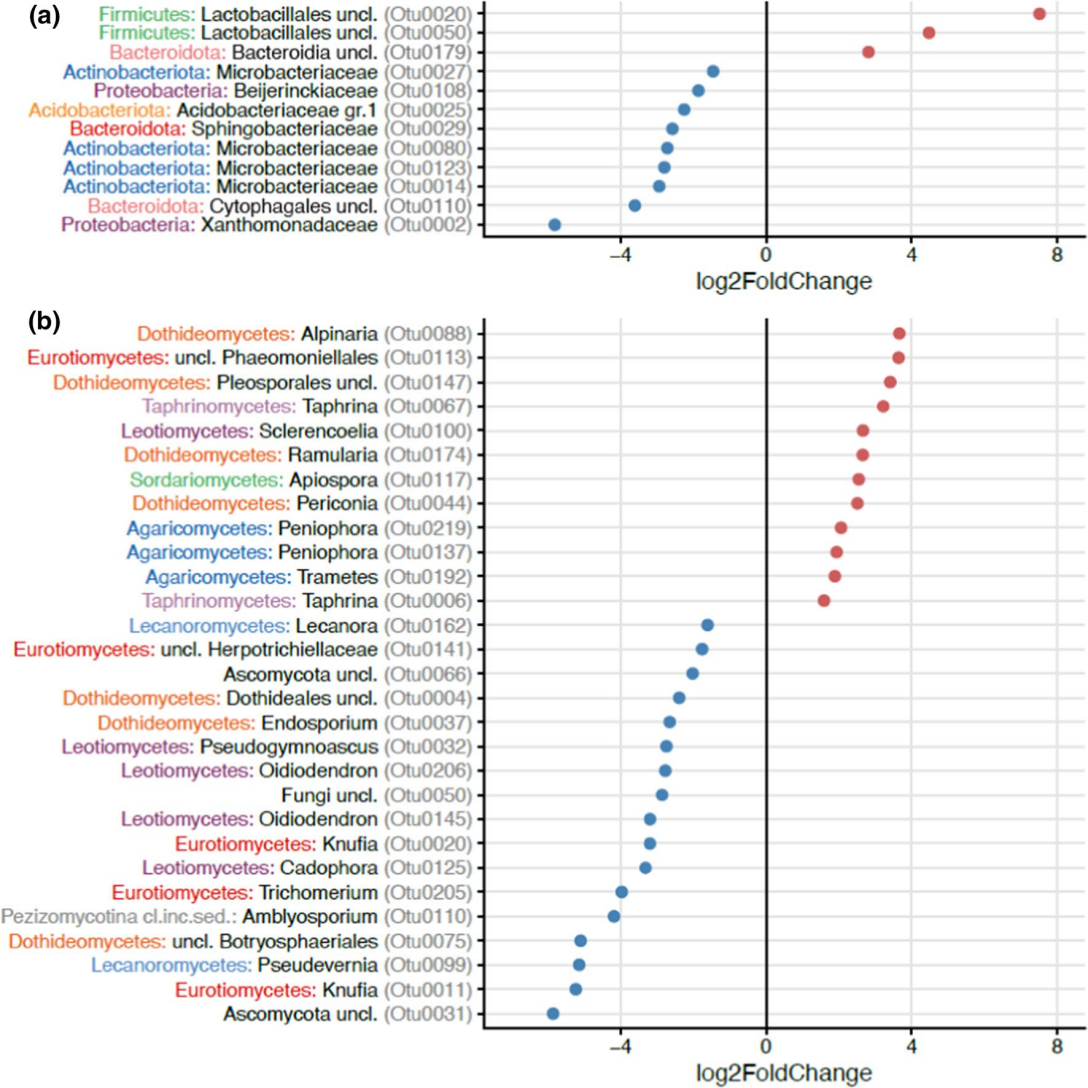
The effect of honeybees on operational taxonomic units (OTUs) of **a** bacteria and **b** fungi in open *Salix phylicifolia* inflorescences (*n* = 47) based on differential abundance analysis with DESeq2. The taxonomic affiliation of the OTUs is shown on the y-axis. The bacterial phyla and fungal classes are marked with colors and the bacterial family and fungal genus are shown in black. OTUs increased by honeybees receive positive values and OTUs relatively more abundant without honeybees receive negative values

**Fig. 2:**
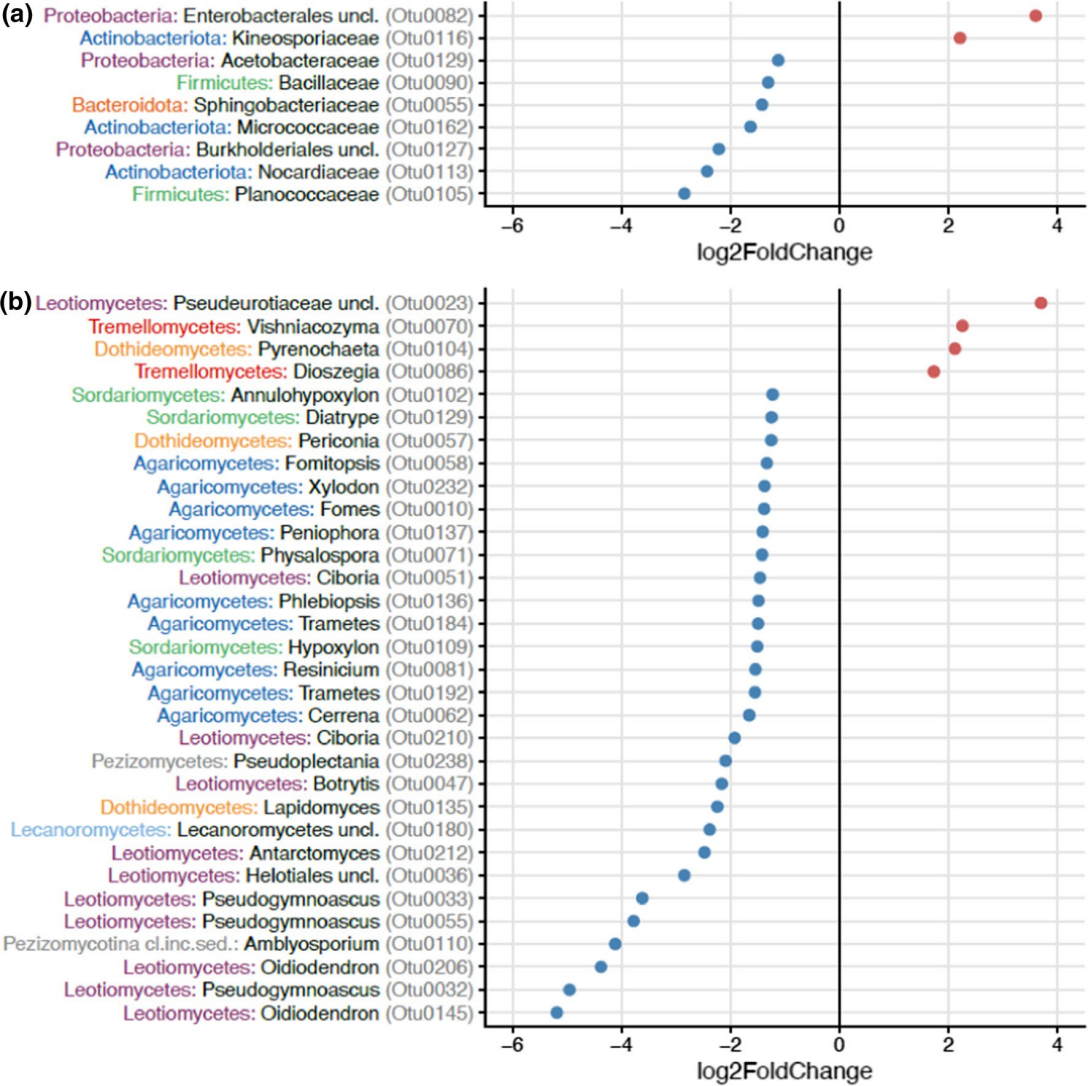
The effect of excluding insect visitation (bagging) on operational taxonomic units (OTUs) of **a** bacteria and **b** fungi in *Salix phylicifolia* inflorescences (*N* = 95) based on differential abundance analysis with DESeq2. The taxonomic affiliation of the OTUs is shown on the y-axis. The bacterial phyla and fungal classes are marked with colors and the bacterial family and fungal genus are shown in black. OTUs increased by bagging receive positive values and OTUs decreased by bagging receive negative values

We further compared which bacteria and fungi differed in relative abundance in open inflorescences between sites where honeybees were foraging and in sites without honeybees (Fig. 1). Honeybees increased the relative abundance of three OTUs, especially the bacterial order Lactobacillales whereas nine OTUs (e.g., Xanthomonadaceae, genus *Xanthomonas*) were relatively more abundant in sites without honeybees when compared to sites close to apiaries (Fig. 1a). Honeybees increased the relative abundance of 12 fungal OTUs from the classes Dothideomycetes (genus *Alpinaria* and order Pleosporales), Eurotiomycetes (order Phaeomoniellales), Taphrinomycetes (genus *Taphrina*), and Leotiomycetes (genus *Sclerencoelia*) among others, whereas 17 OTUs (e.g., Eurotiomycetes (genus *Knufia*), Lecanoromycetes (genus *Pseudevernia*), and Dothideomycetes (Botryosphaeriales)) were relatively more abundant in open inflorescences in sites without honeybees (Fig. 1b). We also compared which bacteria and fungi were differentially abundant between open and bagged inflorescences (Fig. 2). Bagging decreased the relative abundance of seven OTUs that belonged to Planococcaceae, Nocardiaceae, and Burkholderiales among others and increased the abundance of two OTUs from Enterobacterales and Kineosporiaceae (Fig. 2a). For fungi, bagging decreased the relative abundance of 28 OTUs that belonged to Leotiomycetes (genus *Oidiodendron* and *Pseudogymnoascus*) and Pezizomycotina (genus *Amblyosporium*) among others and increased the abundance of four OTUs that belonged to Leotiomycetes (family *Pseudeurotiaceae*), Tremellomycetes (genus *Vishniacozyma* and *Dioszegia*), and Dothideomycetes (genus *Pyrenochaeta*) (Fig. 2b).

Overall, the most abundant bacterial taxa on *Salix phylicifolia* inflorescences belonged to Pseudomonadales followed by Xanthomonadales, Sphingomonadales, Rhizobiales, and Acetobacterales (Fig. S2a). The most abundant fungi belonged to Dothideales, Capnodiales, Lecanorales, and Tremellales (Fig. S2b).

### Microbial richness

**Fig. 3:**
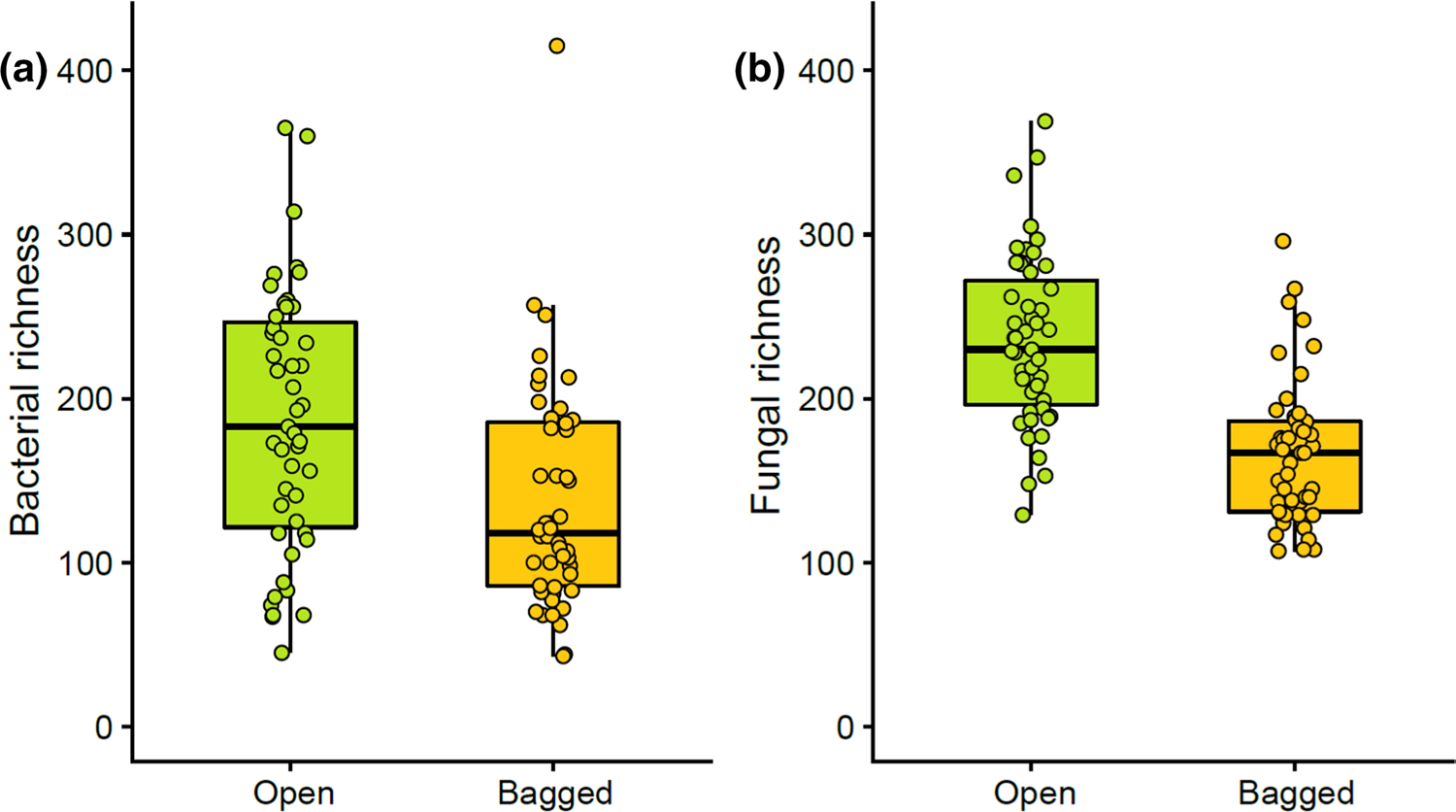
**a** Bacterial and **b** fungal operational taxonomic unit (OTU) richness in open *Salix phylicifolia* inflorescences that insects could visit freely (*n* = 47) and bagged inflorescences (*n* = 48) that were excluded from insect visitation (*N* = 95). The box covers the range from upper to lower quartile, horizontal line shows median, and the whiskers end at minimum and maximum values. Data points are shown as dots

We tested to what extent bagging inflorescences, i.e., excluding insect visits affects inflorescence microbiome. Bagging decreased both bacterial (*df* = 1, *F* = 15.522, *p* < 0.05) (Fig. 3a) and fungal richness (*df* = 1, *F* = 98.747, *p* < 0.05) (Fig. 3b). Site and plant individual were also significant determinants of bacterial and fungal richness on inflorescences (Table S3). Altogether bagging, plant individual and site explained more than 70% of the variation in fungal richness and more than 50% of the variation in bacterial richness (Table 2). The amount of variation in microbial richness explained by insect visitation (i.e., by bagging) was greater in fungi than in bacteria (Table 2).

**Table 2:** The relative importance of the explanatory factors calculated as the amount of variance in operational taxonomic unit (OTU) richness explained by the three experimental factors in a nested ANOVA

	Bacteria (%)	Fungi (%)
Bagging	10	38
Plant individual	21	23
Site	22	15

In each plant, two branches contained open and bagged inflorescences (factor ‘Bagging’). The inflorescences were nested within four *Salix phylicifolia* individuals in each site (factor ‘Plant individual’). Factor ‘Site’ refers to the six study sites (see Table S1)

### Pollen removal by pollinators

**Fig. 4:**
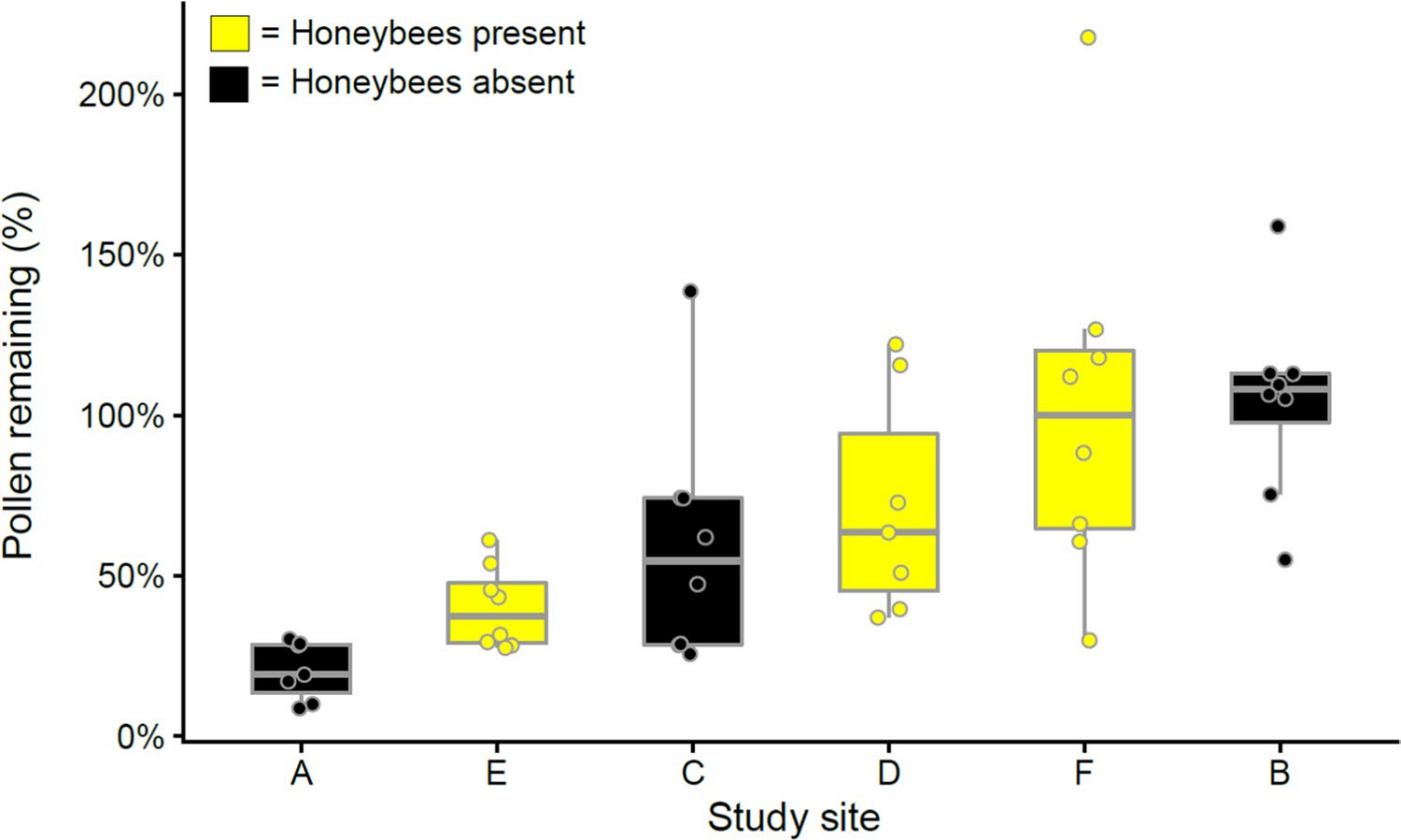
The amount of pollen remaining in the open inflorescences in relation to the pollen in the bagged inflorescences in the same branch of *Salix phylicifolia*. Average percentage of pollen remaining in the inflorescences is shown in the study sites A–F (*n* = 7–8, *N* = 95). Mean values ± standard deviation (SD) are shown

**Fig. 5:**
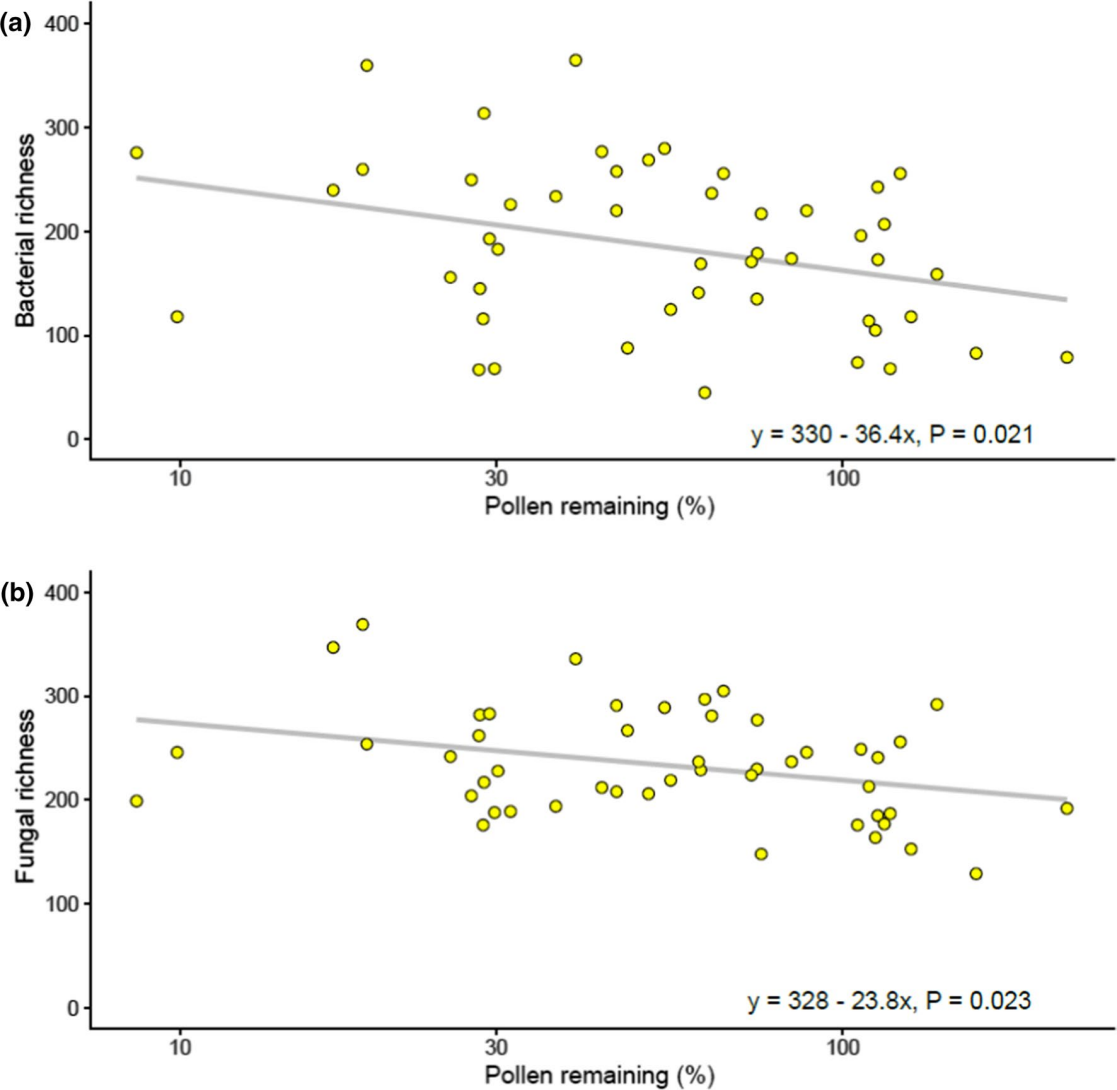
**a** Bacterial and **b** fungal operational taxonomic unit (OTU) richness and amount of pollen remaining in open (not bagged) *Salix phylicifolia* inflorescences. Low values of pollen remaining indicate high pollen removal and high pollinator activity. The *x*-axis (log transformed) shows the average percentage of pollen remaining in the inflorescences (*n* = 47)

We used pollen removal in open inflorescences as a proxy for visitation frequency and related that to OTU richness in open inflorescences. The amount of pollen removed from inflorescences by insect visitors ranged between 0 and 90% (i.e., 10–100% of pollen remained) and was on average 40% (Fig. 4). Site had a significant effect on the amount of pollen removed from inflorescences (*R* = 0.626, *F*_*1,5*_ = 14.032, *p* < 0.05), but this was not due to honeybees (*U* = 345, *p* = 0.240). Pollen removal was correlated with elevated bacterial (*R*^2^ = 0.068, *p* = 0.008) and fungal (*R*^2^ = 0.063, *p* = 0.001) richness on open inflorescences (Fig. 5).

## Discussion

In the present work, we quantified the relative importance of two ecological drivers, insect visitation and plant individual, and collectively the effect of geographical location to the microbial community composition in *Salix phylicifolia* inflorescences. As honeybee farming is an increasing and global trade with unknown effects on natural boreal ecosystems, we teased apart the effect of cultivated honeybees and pollinators in general.

### The microbial fingerprint of cultivated honeybees

Our results are in line with many others showing that insects transmit microbes during floral visit and leave a microbial fingerprint (Ushio et al. 2015) which is unique to the pollinator or taxa, e.g., bumblebees (Brysch-Herzberg 2004; Herrera et al. 2009; Lachance et al. 2001; Morris et al. 2020; de Vega et al. 2009; de Vega and Herrera 2013). In our work, honeybees had a small but significant effect on the overall microbial community structure in open *Salix phylicifolia* inflorescences. In particular, the relative abundance of OTUs from the order Lactobacillales (phylum Firmicutes) was higher in open inflorescences in presence vs absence of honeybees. This agrees with the notion that the Lactobacillales are often associated with pollinators (Chandler et al. 2011; Vasanthakumar et al. 2006) and are an important part of the bee microbiome (Engel et al. 2012; Sabree et al. 2012). Lactobacillales are usually found on nutrient-rich resources and previously shown to be transmitted to flowers (Gaube et al. 2021; McFrederick and Rehan 2019; McFrederick et al. 2017). Our results suggest that Lactobacillales are also spread to wild plants by cultivated honeybees.

Insect visitation does not exclusively enrich floral microbiome but may affect the microbiome composition also by reducing abundance of some microbes. In our work, the relative abundance of OTUs representing the family Xanthomonadaceae was lower in presence of honeybees vs in absence of honeybees. The reduction of members of Xanthomonadaceae in sites without honeybees potentially suggests that the microbes vectored by honeybees negatively affected Xanthomonadaceae abundance by potential intermicrobial interactions (Trivedi et al. 2020). However, this interaction should be quantitatively and experimentally verified.

### Insect visitation increases bacterial and fungal richness and affects community composition

The bacterial and fungal richness in *Salix phylicifolia* inflorescences were of the same magnitude, which is surprising as globally the kingdom Fungi constitute only 7% of the richness in Bacteria (Larsen et al. 2017). This suggests that flowers may be a particularly favorable habitat for fungi. Some of the fungi we discovered in *Salix phylicifolia* inflorescences were unidentified. Several studies have identified novel species of fungi isolated from tropical flowers (Groenewald et al. 2011; Ottesen et al. 2013; Rosa et al. 2007) and the same most likely applies to temperate and boreal plants. Flowers seem to be a hotspot of fungal species richness and future studies should evaluate the ecological ramifications of this ephemeral but rich community.

Insect visitation increased more fungal than bacterial richness. This suggests that the floral fungal community is particularly dependent on insect-vectored dispersal. This is in line with the fact that many of the plant diseases transmitted through floral visitors are fungal pathogens (Batra and Batra 1985; Jennersten 1988). We found that representatives of Taphrinomycetes were relatively more abundant in honeybee sites. Members of *Taphrina*, the only genus in the family Taphrinaceae, parasitize on plants and cause witch's brooms and catkin curl diseases in certain flowering plants (Mix 1935). In a recent study, honeybees participated to microbial assembly of the seed through pollination, and thus, microbes that arrive as a result of floral visits can influence plant fitness (Prado et al. 2020). Altogether, the importance of the rich floral fungal microbiome vectored by insects on plant reproduction and on pollinators warrants further research.

Because previous research has been nearly entirely focused on microbes in floral nectar (de Vega et al. 2009; Herrera et al. 2009; Jacquemyn et al. 2013; Lachance et al. 2001), the effect of pollinators on the microbial richness in other flower organs or entire inflorescences may be greater than previously thought. For example, Pozo et al. (2012) investigated the main factors responsible for yeast frequency and species richness in two Spanish flowering plant species and found that the highest fungal species richness was in corolla samples and the lowest in pollen and nectar. This is supported by the work by Russell et al. (2019) who showed that the corolla of *Mimulus spp.* received the most microbes during insect visitation.

In our field work, insect visitation increased the abundance of taxonomically diverse bacteria. Many of these bacteria such as Firmicutes and Actinobacteria, have been isolated previously in flowers (Aizenberg-Gershtein et al. 2013; Fridman et al. 2012; Fürnkranz et al. 2012; Jacquemyn et al. 2013; Shade et al. 2013). Because the altered bacterial groups are common and present in flowers and the environment, they are difficult to associate specifically with insect visitation, although the results suggest that. In terms of fungi, some taxa that increased with insect visitation are probably generalists colonizing various substrates such as the representatives of the genus *Oidiodendron* (Myxotrichaceae, Ascomycota). Members of *Oidiodendron* have been discovered in various substrates such as soil and feathers (Udakawa and Uchiyama 1998) and termite nests (Roose-Amsaleg et al. 2004). The fungal OTUs that increased along with the exclusion of insects include basidiomycetous yeasts in the Bulleribasidiaceae such as *Vishniacozyma* and *Dioszegia* that have been shown to be important members of the fungal community in nectar previously (Peter et al. 2017; Moubascher et al. 2018). This suggests that in absence of pollinator visits, nectar accumulates in the inflorescences (Varga et al. 2013) and may facilitate the growth of these yeasts in particular.

### Plant individual and site: not out of sight

Floral microbiome is largely a subset of foliar microbiome (Wei and Ashman 2018; Massoni et al. 2019), suggesting that foliar surfaces are the main source of floral microbiome. Rain has been shown to scavenge and deposit microbes on surfaces (Allard et al. 2020), and consequently, the weather during measurement period could cause variation in floral microbiomes. However, *Salix phylicifolia* blooms before leaf flush and there was no precipitation during the present investigation period. The high proportion of microbial community composition due to the site (15% in fungi and 22% in bacteria) in the current work highlights the importance of dry deposited microbes originating from local soil and surrounding vegetation as sources of *Salix phylicifolia* inflorescence microbiome. Geographic variation within species in floral microbiome has not been quantified previously. However, locality has been shown to be the most important driver for plant microbiome in general and overriding that of the plant individual (Hamonts et al. 2018; Brown et al. 2020) or even species (Zhang et al. 2020).

Previous studies on crop plants have shown that plant individual has a minor (≤ 5% variation explained) effect on plant microbiome (Edwards et al. 2015). In our boreal ecosystems, plant individual was a major determinant of the inflorescence microbiome (> 20% of variation explained) and had an equal effect to that of site. This result may be due to the relatively simple microbiomes in flowers in comparison to rhizosphere (Berendsen et al. 2012; Bron et al. 2012; Mendes et al. 2011; Raaijmakers et al. 2009), and in the early season microbiomes in comparison to those later in the season (Shade et al. 2013), which highlighted the role of plant individual in our work. However, a few studies on wild plants suggest that the plant individual may be an important driver of wild plant microbiome composition. For example, genotype explained 12% of microbiome variation in *Populus trichocarpa* rhizosphere (Veach et al. 2019).

### Pollen removal by insects

Pollinators forage in flowers to acquire resources and provide opportunities for plants to disperse pollen grains among flowers. Pollen houses a part of the plant microbiome (Manirajan et al. 2016) and contains microbes both inside (Bristow and Martin 1999) and on the surface (Fürnkranz et al. 2012; Manirajan et al. 2016). Thus, pollen can be an important but unappreciated vector for microbes to interact between both plants and pollinators (Rebolleda-Gómez and Ashman 2019). In our study, insect visitation (quantified as pollen removal) increased microbial richness in the inflorescences. Part of the increase could originate from additions of insect microbiome (McFrederick et al. 2017; Prado et al. 2020) on inflorescences and part from the dispersal of microbes between inflorescences. The amount of pollen removed from inflorescences by insect visitors ranged considerably between the study sites in the present study, but the variation was not explained by presence of honeybees. This may be due to variability in the presence, abundance and activity of insect visitors in general between sites as the site itself explained pollen removal. Pollen is a vital resource for honeybees and bumblebees that both feed the next generation with the protein-rich pollen (Dötterl and Vereecken 2010). Only half of the *Salix phylicifolia* in a given population produce pollen as female plants produce only nectar (Elmqvist et al. 1988). Furthermore, pollen is not replenished by the plant unlike nectar and, therefore, exploitative competition for pollen may be more intense than for nectar. However, the fact that honeybees did not increase pollen removal from *Salix phylicifolia* inflorescences suggests that, at least in the present rural sites, competition for pollen between honeybees and other pollinators during a critical moment in spring was not significant. Resource competition between commercial and wild pollinators has been suggested as one of the reasons for global decline in wild pollinators (Geslin et al. 2010); however, few studies explicitly address it. It should be noted that we did not quantify the visitation rate by honeybees and future studies are needed to estimate the sustainable number of beehives in a given landscape.

## Conclusions

Floral microbiome is an understudied and underexplored ecological factor that affects pollinator attraction (Peach et al. 2021; Russell and Ashman 2019) and thus pollination (Herrera et al. 2013; Herrera and Medrano 2017) and plant reproduction (Yang et al. 2019; Schaeffer and Irwin 2014; Vannette et al. 2013), but also has consequences on the pollinators (Fürst et al. 2014; Graystock et al. 2015) with presently unknown ecological dimensions. Wild pollinator populations are globally declining at the same time as honeybee farming and trading is increasingly intensive. In our work, cultivated honeybees changed the microbiome in a central floral resource shared with wild pollinators, most notably bumblebees. Filling current knowledge gaps of these ecological interactions is a key for predicting the ecosystem effects of honeybee farming. Some of the unidentified fungi may live on the surface of pollen, because we exclusively surveyed the inflorescences in male *Salix phylicifolia*. Data from pollen microbiomes are notably missing which indicates how poorly the microbial community in flowers is known. Our work showed that the fungal diversity in inflorescences is dependent on the floral visitors more than that of bacteria. Quantifying the effect of the changes in floral microbial community on plant reproduction and on pollinators is a challenge for future research.

## Supplementary Information

Below is the link to the electronic supplementary material.

Supplementary file1 (DOCX 572 KB)

## Data Availability

The sequence data has been submitted to NCBI under BioProject accession PRJNA776874. The R code and data files are available at https://github.com/helijuottonen/elsiwillow.
