# Supplementary material for: Honeybees affect floral microbiome composition in a central food source for wild pollinators in boreal ecosystems
Source: Oecologia. 2022 Nov 24;201(1):59–72. doi: 10.1007/s00442-022-05285-7 (PMC9813210; doi:10.1007/s00442-022-05285-7)
Supplement: Supplementary file 1 — Supplementary file1 (DOCX 572 KB) [file 442_2022_5285_MOESM1_ESM.docx]

**Supplementary Material**

Title: Honeybees affect floral microbiome composition in a central food source for wild pollinators in boreal ecosystems

**Authors: Elsi Hietaranta^*^, Heli Juottonen & Minna-Maarit Kytöviita**

**Department of Biological and Environmental Science, University of Jyväskylä,**

**P.O. Box 35, 40014 University of Jyväskylä, Finland**

***corresponding author: elsi.h.hietaranta@student.jyu.fi**

**Table S1** The characteristics of the six study sites and the dates of bagging and sampling of the inflorescences. Number of beehives refers to the number of beehives in the nearest apiary

| **Study site** | **Coordinates** | **Honeybees** | **Number of beehives** | **Distance to the closest beehives** | **Net bags attached** | **Samples collected** |
| --- | --- | --- | --- | --- | --- | --- |
|  |  |  |  |  |  |  |
|  |  |  |  |  |  |  |
| A | 62°46' N : 25°18' E | No |  | > 3.5 km | 24-Apr-2019 | 28-Apr-2019 |
| B | 61°54' N : 26°02' E | No |  | > 10 km | 16-Apr-2019 | 23-Apr-2019, 29-Apr-2019 |
| C | 62°01' N : 26°17' E | No |  | > 10 km | 16-Apr-2019 | 25-Apr-2019, 29-Apr-2019 |
| D | 62°46' N : 25°23' E | Yes | 7 | 100-200 m | 17-Apr-2019 | 28-Apr-2019 |
| E | 62°03' N : 26°08' E | Yes | 4 | 50-100 m | 16-Apr-2019 | 23-Apr-2019 |
| F | 61°49' N : 25°56' E | Yes | 6 | 10-200 m | 16-Apr-2019 | 23-Apr-2019, 25-Apr-2019 |


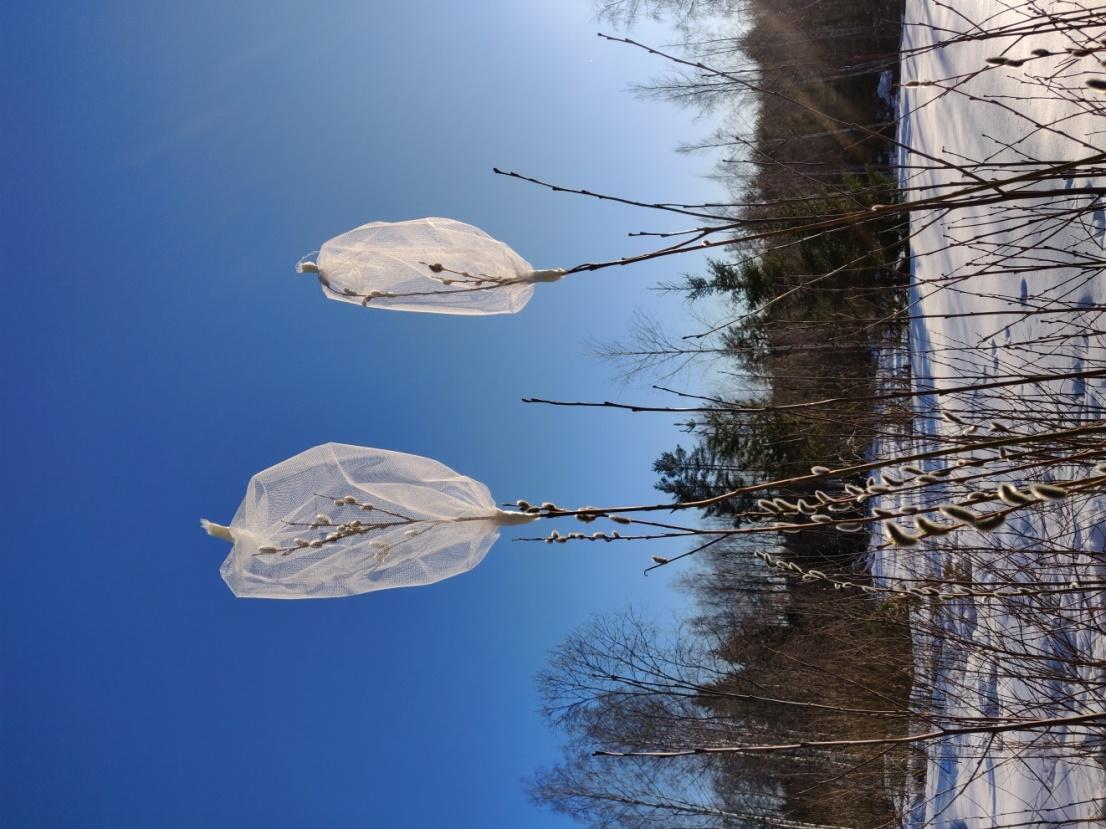


**Fig. S1** Willow branches enclosed with a net bag to exclude insect visits on *Salix phylicifolia* inflorescences. Samples that were visited by insects were collected from inflorescences in branches next to net bags

**Table S2** PERMANOVA statistics on effect of *Salix phylicifolia* individual (*n* = 4) on the structure of bacterial and fungal community composition in the inflorescences (*N* = 95). Significance codes are *p* *≤* 0.001 *= ****, *p* *≤* 0.01 *= ***, *p* *≤* 0.05 *= **

| **Site** | **Bacteria** | | | | | **Fungi** | | | | |
| --- | --- | --- | --- | --- | --- | --- | --- | --- | --- | --- |
|  | *df* | *SS* | *R^2^* | *F* | *p* | *df* | *SS* | *R^2^* | *F* | *p* |
| A | 3 | 1.22 | 0.55 | 4.96 | 0.002 ** | 3 | 1.33 | 0.37 | 2.40 | 0.001 *** |
| B | 3 | 1.08 | 0.24 | 1.29 | 0.167 | 3 | 0.88 | 0.35 | 2.14 | 0.004 ** |
| C | 3 | 1.26 | 0.41 | 2.60 | 0.021 * | 3 | 0.86 | 0.34 | 1.91 | 0.002 ** |
| D | 3 | 1.59 | 0.43 | 2.98 | 0.001 *** | 3 | 0.69 | 0.27 | 1.50 | 0.053 |
| E | 3 | 1.00 | 0.32 | 1.91 | 0.033 * | 3 | 1.22 | 0.53 | 4.59 | 0.001 *** |
| F | 3 | 1.04 | 0.37 | 2.30 | 0.021 * | 3 | 0.61 | 0.36 | 2.23 | 0.009 ** |


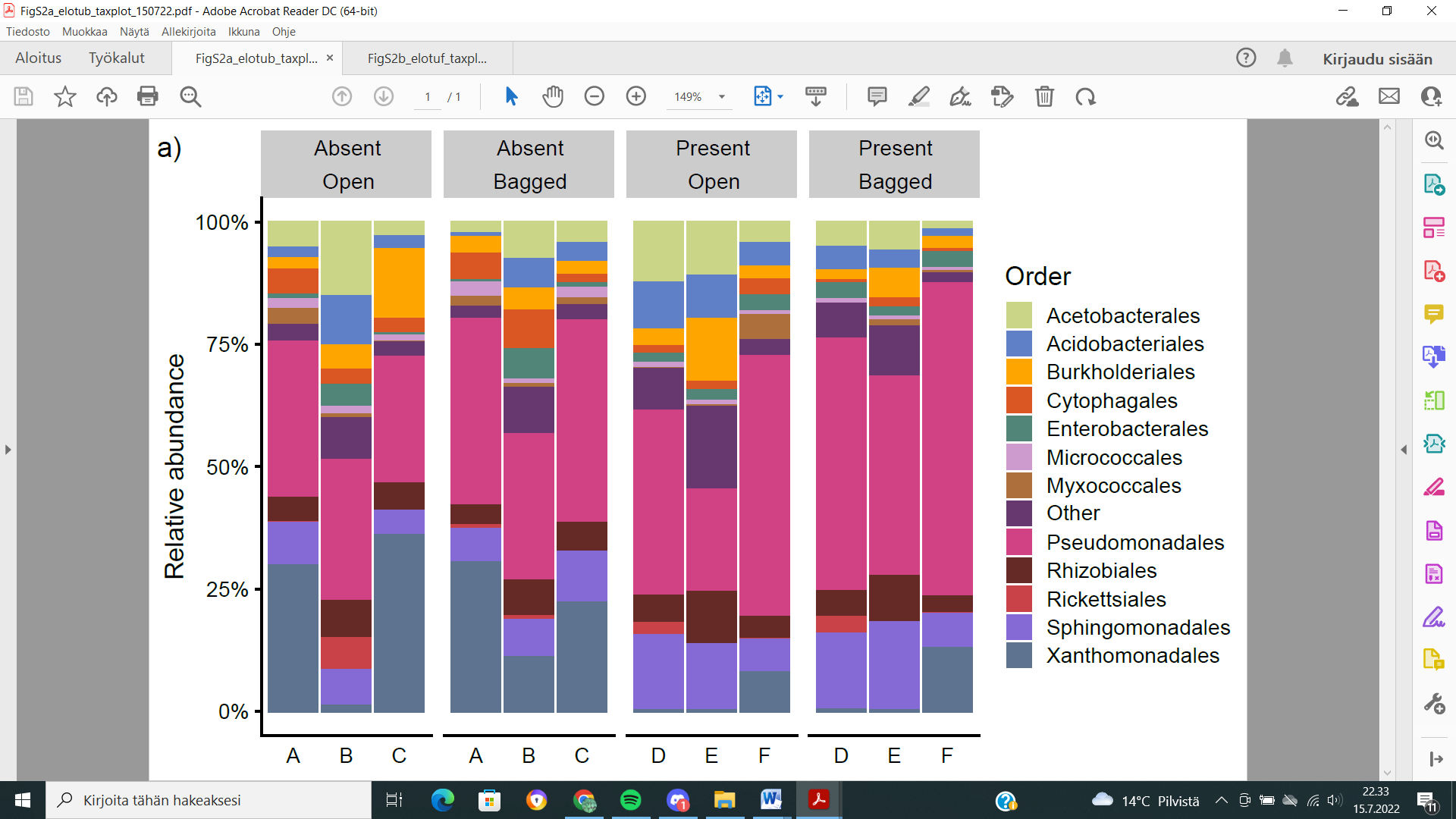


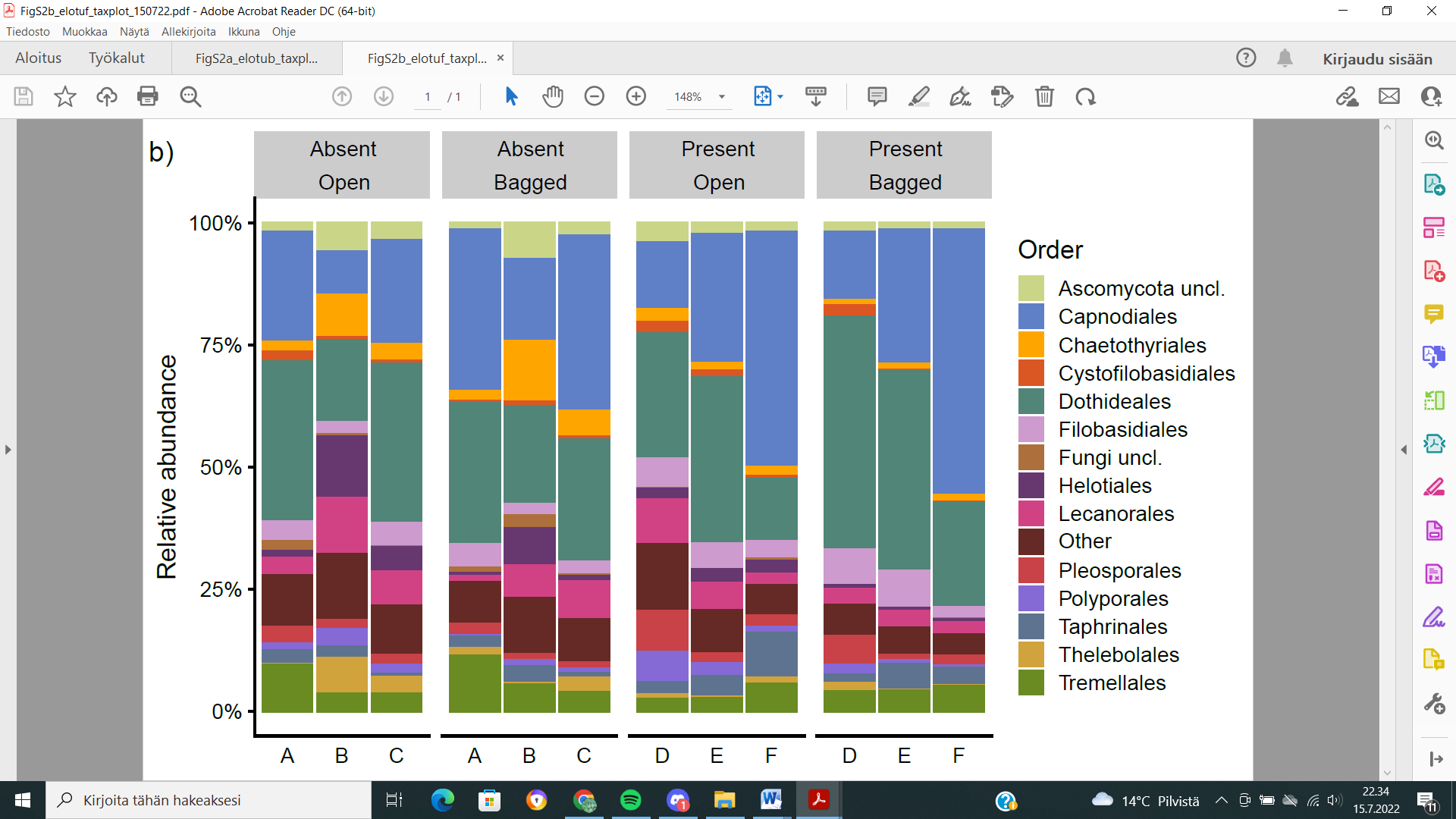


**Fig. S2** The relative abundance of **a** bacterial orders and **b** fungal classes in the six study sites A–F (*n* = 15–16) divided by honeybees (absent/present) and treatment (open/bagged). Parallel samples from each site are pooled (*N* = 95). ‘Other’ represents bacterial orders and fungal classes which relative abundance is < 1%

**Table S3** Bacterial and fungal operational taxonomic unit (OTU) richness by study site and treatment. Site affected significantly bacterial (*df* *=* 5, *F* *=* 6.789, *p* *<* 0.05) and fungal (*df* *=* 5, *F* *=* 8.103, *p* *<* 0.05) richness on inflorescences. ‘Site’ refers to the six study sites (see Table S1). ‘Treatment’ refers to the inflorescences that were either bagged with a mesh bag to exclude pollinators (*n* = 48) or left without the mesh bag (open) (*n* = 47). ‘Honeybees’ refers to the presence/absence of honeybees in the sites. SD means standard deviation

| **Site** | **Treatment** | **Honeybees** | **mean OTU richness** | | | | **SD** | |
| --- | --- | --- | --- | --- | --- | --- | --- | --- |
|  |  |  | | **bacteria** | **fungi** | **bacteria** | | **fungi** |
| A | open | no | 213 | | 248 | | 76 | 56 |
|  | bagged |  | 144 | | 166 | | 51 | 47 |
| B | open | no | 142 | | 185 | | 56 | 41 |
|  | bagged |  | 104 | | 140 | | 39 | 27 |
| C | open | no | 126 | | 257 | | 51 | 25 |
|  | bagged |  | 134 | | 193 | | 63 | 51 |
| D | open | yes | 218 | | 222 | | 56 | 48 |
|  | bagged |  | 149 | | 133 | | 52 | 26 |
| E | open | yes | 203 | | 227 | | 71 | 34 |
|  | bagged |  | 153 | | 155 | | 84 | 24 |
| F | open | yes | 117 | | 194 | | 61 | 41 |
|  | bagged |  | 74 | | 155 | | 23 | 33 |
